# Metabolic reprogramming in malignant A375 cells treated with a ruthenium (II) complex: insights from GCxGC-TOF/MS metabolomics

**DOI:** 10.1007/s11306-025-02221-7

**Published:** 2025-01-20

**Authors:** Francis Adu-Amankwaah, Ayesha Hussan, Gershon Amenuvor, Vuyo Mavumengwana, Lungile Sitole

**Affiliations:** 1 Department of Biochemistry, Faculty of Science, University of Johannesburg, Johannesburg, 2006 South Africa; 2 South African Medical Research Council Centre for Tuberculosis Research, Division of Molecular Biology and Human Genetics, Faculty of Medicine and Health Sciences, Stellenbosch University, Stellenbosch, 7505 South Africa; 3 Department of Chemistry, Faculty of Science and Computational Sciences, Kwame Nkrumah University of Science and Technology, Kumasi, Ghana

**Keywords:** Metabolomics, Cellular, Malignant melanoma, Two-dimensional gas chromatography time-of-flight mass spectrometry (GCxGC-TOF/MS), Ruthenium complexes

## Abstract

**Introduction:**

Melanoma is an aggressive form of cancer characterised by its high metabolic adaptability that contributes to drug resistance. To this end, ruthenium complexes have emerged as a promising class of compounds in the discovery of cancer drugs due to their unique chemical properties and potential to overcome some of the limitations of conventional chemotherapy. In our previous study, we synthesised, characterised, and performed cytotoxicity tests of a ruthenium (II) complex (GA113) against the malignant A375 melanoma cell line. Our previous findings revealed favourable cytotoxicity, with an IC_50_ value of 8.76 µM which formed the basis current study.

**Objective:**

Elucidate the metabolic mechanism of GA113 in malignant A753 melanoma cells.

**Method:**

A two-dimensional gas chromatography time-of-flight mass spectrometry (GCxGC-TOF/MS) cellular metabolomics approach was used, and univariate and multivariate statistical methods were applied to the metabolomics data.

**Results:**

33 metabolites were identified as significant discriminators between GA113-treated and untreated A375 melanoma cells. Changes in 19 of these 33 metabolites were mapped to pantothenate and coenzyme A biosynthesis, citrate cycle, cysteine and methionine metabolism, arginine and proline metabolism, and alanine, aspartate, and glutamate metabolism.

**Conclusion:**

These findings suggest that GA113 exerts its anticancer effects by disrupting essential metabolic pathways in melanoma cells, which presents a promising therapeutic avenue to target melanoma metabolism.

**Supplementary Information:**

The online version contains supplementary material available at 10.1007/s11306-025-02221-7.

## Introduction

Melanoma remains one of the most aggressive and lethal forms of skin cancer, accounting for the majority of skin cancer-related deaths worldwide (Sadeq et al., 2023). While the advent of targeted therapies, along with immune checkpoint inhibitors, has marked significant progress in treating advanced melanoma, the long-term efficacy of these interventions remains limited (Sadeq et al., 2023; Ziogas et al., 2023). A substantial proportion of patients do not respond or eventually develop therapeutic resistance, leading to disease relapse and progression within a short period of remission (Rambow et al., 2019). This resistance, often driven by genetic heterogeneity and tumour plasticity, highlights the need for novel therapeutic approaches that can address the metabolic and adaptive mechanisms utilised by melanoma cells (Rambow et al., 2019).

Ruthenium complexes have emerged as promising candidates in cancer treatment due to their unique chemical and biological properties (Thota et al., 2018). Unlike traditional platinum-based drugs, ruthenium complexes exhibit lower toxicity to non-malignant cells and greater selectivity for malignant cells (Lee et al., 2020; Sun et al., 2021). Ruthenium has various oxidation states, enabling it to participate in diverse biochemical interactions, and it can mimic iron, allowing it to exploit the iron transport pathways used by cancer cells (Lu et al., 2022). Therefore, researchers are actively exploring ruthenium-based compounds for their potential to offer effective and less toxic alternatives to conventional cancer therapies (Lin et al., 2018; Sun et al., 2021; Thota et al., 2018). The mechanism of action of ruthenium-based anticancer drugs is understood to some extent. However, to optimise drug design (enhance selectivity and efficacy), it is key to obtain comprehensive molecular signatures that inform how these anticancer drugs target unique metabolic processes in cancer cells.

Cell culture metabolomics has gained traction and is currently being used to decipher the cellular metabolic alterations induced by metallodrugs in various cancer cell lines (De Castro et al., 2019; Castro et al., 2022; Galvez et al., 2019; Ghini, 2024; Halama, 2014; Zhang et al., 2022; Zong et al., 2018). Previously, our group synthesised, characterised, and tested the cytotoxicity of a series of ruthenium complexes containing a bis-amino phosphine ligand (Engelbrecht et al., 2018; Hussan et al., 2023). Among the complexes, GA113 was the most potent, with an IC_50_ < 10 µM in malignant A753 melanoma cells. Using a nuclear magnetic resonance (NMR) metabolomics approach, Hussan et al. (2023) investigated the effect of GA113 on the metabolic profile of the A375 cells. In this study, we expand on this previous NMR investigation by applying a two-dimensional gas chromatography time-of-flight mass spectrometry (GCxGC-TOF/MS) metabolomics approach to obtain a more comprehensive molecular signature of A375 cells treated with GA113.

Studies have reported on the application of GC-MS-based metabolomics to investigate the mechanism of action of metal-based anticancer drugs (Granit et al., 2022; Obrist et al., 2018; Pyo et al., 2008; Soares et al., 2022). However, only a limited number of studies have used GCxGC-TOF/MS. Although one-dimensional GC-MS can offer excellent resolution, some applications require increased resolution power. To this end, GCxGC-TOF/MS is increasingly being used in cutting-edge research as a powerful approach that offers enhanced separation, sensitivity, and resolution for complex biological samples (Winnike et al., 2015). In the context of anticancer drug design and development, GCxGC-TOF/MS metabolomics analysis can reveal off-target effects and potential biomarkers for response, which are valuable for predicting and minimising toxicity (Halama, 2014). Additionally, GCxGC-TOF/MS metabolomics can contribute to our understanding of how potential anticancer drugs interact with cellular redox systems, possibly allowing adjustments that fine-tune their oxidative stress-inducing effects and reduce adverse reactions in healthy cells (Halama, 2014). This study used a GCxGC-TOF/MS metabolomics approach to investigate the possible metabolic mechanism of action of GA113 in a human malignant A375 cell line.

## Materials and methods

### Complex synthesis

**Fig. 1:**
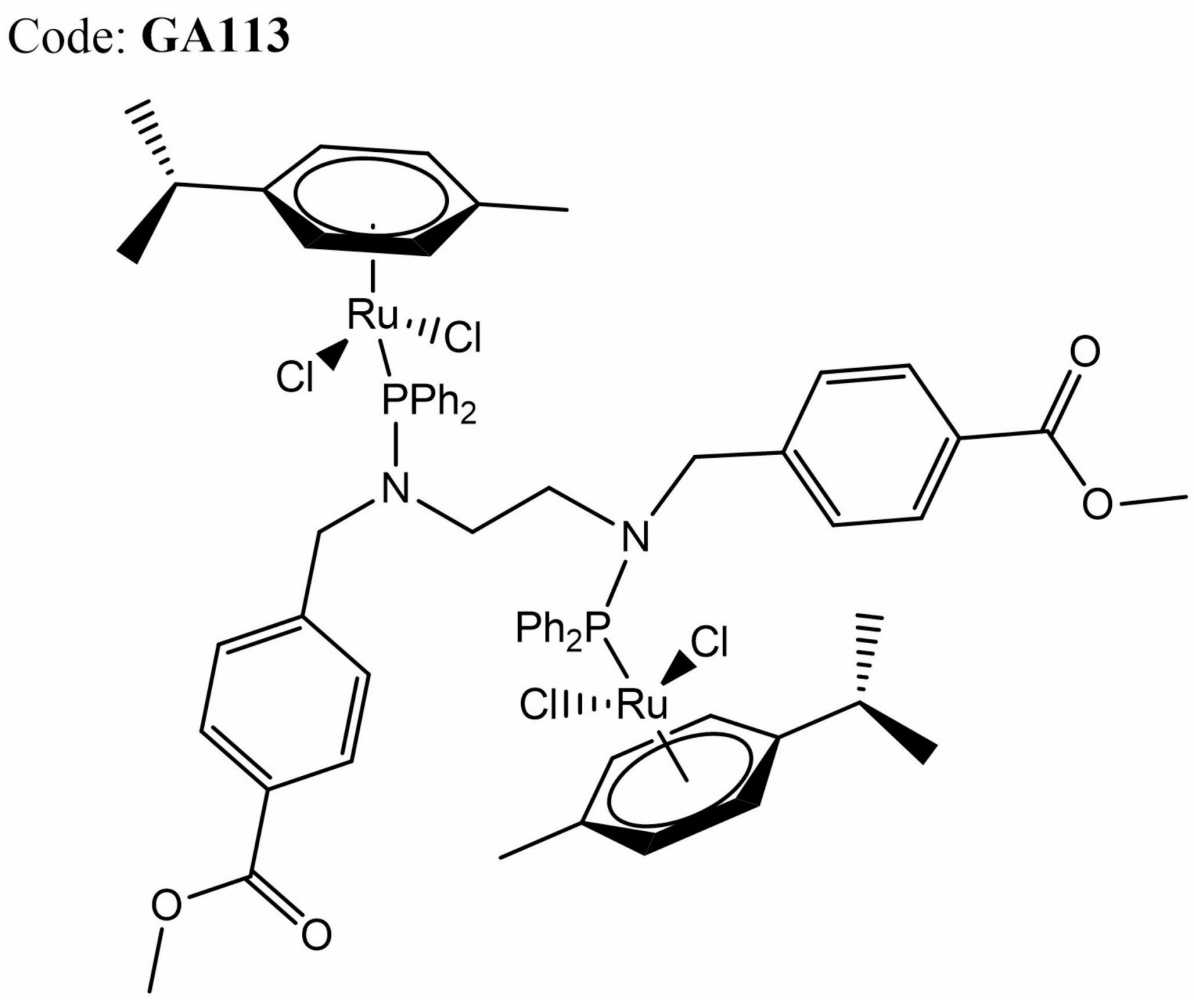
Structure of (ŋ^6^-p-cymene)Ru(Cl_2_)(PPh_2_(p-MeBenzoate)NCH_2_CH_2_N(p-MeBenzoate)PPh_2_)(Cl_2_)Ru(ŋ^6^-p-cymene) (GA113)

The bimetallic complex (GA113; Fig. 1) investigated here was synthesised according to previously reported methods and provided satisfactory analytical data (Engelbrecht et al., 2020; Hussan et al., 2023).

### Cell culture

Human malignant A375 melanoma cells were obtained from Prof. Kidzeru at the Hair and Skin Research Laboratory, Division of Dermatology, Department of Medicine, Faculty of Health Sciences, Groote Schuur Hospital, University of Cape Town, South Africa. Cells were cultured in Dulbecco’s modified Eagle medium (DMEM) (Hyclone™, GE Healthcare and Life Sciences, USA) with high glucose, supplemented with 10% foetal bovine serum (FBS) (Sigma, St. Louis, MO, USA) and 1% penicillin/streptomycin/amphotericin B solution (Sigma, St. Louis, MO, USA). Cultures were maintained in 75 cm² flasks within a sterile Heracell™ VIOS 160i incubator, providing a humidified atmosphere of 37 °C and 5% CO_2_. The culture medium was replaced with fresh-supplemented DMEM (20 mL) the following day. The cells were subcultured every two days.

### Cytotoxicity assay

The treatment procedure and cytotoxicity evaluation of GA113 against the malignant A375 cells are reported in our previous publication (Engelbrecht et al., 2020). The IC_50_ value (8.76 µM ± 1.37 µM) was calculated in Microsoft Excel from the dose-response curve using nonlinear regression analysis (Engelbrecht et al., 2020). The light microscopy and flow cytometry data generated from our previous study also showed that, after a 24-hour treatment exposure, GA113 induced obvious cell death in A375 cells (Engelbrecht et al., 2020; Hussan, 2021).

### Cellular viability

The concentration of GA113 used in this study was selected based on the IC_50_ value obtained from our previous publication (Engelbrecht et al., 2020). Initially, three concentrations were selected: low concentration (2.5 µM); middle concentration (5 µM); and IC_50_ concentration. Before the GCxGC-TOF/MS analysis, the extent of cell death, following treatment with each concentration, was assessed using the Trypan blue exclusion assay (Thermofisher Scientific, USA). Based on the Trypan blue results (Table 1), we selected a concentration of 5 µM for this study, ensuring minimal cytotoxicity (cell viability 80%). This concentration was selected to avoid metabolite changes resulting from reduced cell viability.

**Table 1:** Effects of different concentrations of GA113 on cellular activity of A375 for 24 h

GA113 concentration (µM)	Cell viability (%) (*n* = 3; mean ± SD)*
0 (untreated cells)	95 ± 1
2.5	91.67 ± 2.08
5	80 ± 4.36
8.76 (IC_50_)	50%
10	45.67 ± 4.04

*SD– standard deviation. Published in Engelbrecht et al. (2020) and Hussan (2021)

### Treatment for GCxGC-TOF/MS metabolomics

A375 melanoma cells were cultured in six-well plates at a concentration of 5 × 10^5^ cells/mL, with 5 mL per well, resulting in a total of 2.5 × 10^6^ cells per well. Once the cells reached a confluency of 70–80%, the cells were treated with GA113 at 5 µM for 24 h. For the control cells, a fresh medium without treatment was added. Following treatment, the cells were washed three times with ice-cold phosphate-buffered saline (PBS) (1X). To quench cellular metabolism, 1 mL of ultra-pure ice-cold methanol (Romil Pure Chemistry, UK) was added to each well. The cells were subsequently harvested by scraping the bottoms of the plates using a cell scraper and transferred to Eppendorf tubes. The harvested cell samples were snap frozen in liquid nitrogen, labelled, and stored at -80 °C until shipped on dry ice to the Centre for Human Metabolomics at North-West University, South Africa, for further analysis. The same procedure was meticulously used for all samples to minimise experimental variability. Three independent assays were performed.

### Quality control samples

A master pooled quality control (QC) sample was created by combining small volumes from all collected cell samples. Several smaller aliquots were prepared from this master QC sample. These QC aliquots were run as the first, middle, and last samples in each GCxGC-TOF/MS batch. To evaluate the quality of the obtained metabolomics data, the repeatability of the instrument and extraction process was assessed by evaluating these QCs.

### Sample preparation for metabolite extraction and analysis

For metabolite extraction, all experimental samples and quality control (QC) samples were dried under a gentle stream of nitrogen gas to remove the methanol. A single-phase extraction method was used, where 50 µL of internal standard 3-phenylbutyric acid (Sigma-Aldrich, USA) was added to each sample vial, along with 1 mL of the extraction solvent, which was composed of chloroform, methanol, and water in a 1:3:1 ratio (Burdick and Jackson, Honeywell International Inc., USA). Additionally, a 3 mm tungsten bead (Retsch GmbH & Co. KG, Germany) was included in each tube.

The samples were placed in a vibration mill and homogenised at 30 Hz for 5 min, followed by centrifugation at 10,000 × g for 5 min at 4 °C. The supernatant was transferred to glass GC vials and dried under a gentle stream of nitrogen at 40 °C for 20–30 min. For derivatization, each dried sample received 50 µL of methoxyamine hydrochloride (Sigma Aldrich, Germany) and was incubated at 50 °C for 90 min. Subsequently, 40 µL of N, O-bis(trimethylsilyl)trifluoroacetamide (BSTFA) with 1% trimethylchlorosilane (TMCS) (Sigma Aldrich, USA) was added, and the samples were incubated at 60 °C for 60 min. The derivatized samples were then transferred to 0.25 mL inserts in clean GC sample vials and analysed using GCxGC-TOF/MS.

### GCxGC-TOF/MS analysis

Analysis was carried out using a Pegasus 4D GCxGC-TOF/MS (Leco Corporation, USA), which consisted of an Agilent 7890 A GC (Agilent, Georgia, USA) coupled to a time-of-flight mass spectrometer (TOF/MS) (Leco Corporation, USA) equipped with a Gerstel Multi-Purpose Sampler (MPS) (Gerstel GmbH & Co. KG, Germany). The system was equipped with a cryogenic cooler. The first separation was carried out on an Rxi-5Sil-MS primary column (30 m x 0.25 mm x 0.25 µM) (Restek, South Africa) and the second separation was done on an Rxi-17 secondary column (1.380 m x 0.25 mm x 0.25 µM) (Restek, South Africa). Helium was used as the carrier gas with a constant flow rate of 1 mL/min. One microlitre of each sample, in a random injection order, was injected with a split ratio of 1:10. The samples were injected with a constant inlet temperature of 270 °C. The primary GC oven temperature was initially programmed at 70 °C for 2 min, then increased at a rate of 4 °C/min to a final temperature of 300 °C, where it was held for 2 min. The secondary oven was initially programmed at 85 °C for 2 min, then increased at 4.5 °C/min to a final temperature of 300 °C, where it was held for 4.5 min. The modulator was programmed with an initial temperature of 100 °C for 2 min after which it was increased by 4 °C/min to a final temperature of 310 °C, where it was held for 12 min. Cryomodulation and a hot pulse of nitrogen gas of 0.5 s every 3 s were used to control the effluent that emerged from the column into the secondary column. There was a solvent delay for the first 350 s of each run. The transfer line temperature was held at 280 °C and the ion source temperature at 200 °C for the duration of the run. The acquisition voltage of the detector was 150 V, and electron ionisation spectra were recorded at 70 eV. Data were acquired at 200 spectra/sec. The MS covered a m/z range of 50–800.

### GCxGC-TOF/MS data processing

ChromaTOF software version 4.50 (Leco Corporation, USA) was used for peak identification, mass spectral deconvolution, and peak alignment at a signal ratio of 100, with a minimum of three apexing peaks. Compounds were identified by comparing their mass fragmentation patterns and their retention times with commercially available National Institute of Standards and Technology (NIST) spectral libraries (named ‘mainlib’ and ‘replib’) after using a level 3 identification method published by Schymanski et al. (2014). Peaks identified in the system as blank samples were deleted from the dataset when these peaks had an intensity of one-third or more of that detected for the same peak in the analytical samples, as these were contaminants. The same experimental procedure was performed on the extraction blanks– an initially empty sample vial containing no biological specimen. Thereafter, the compound areas were normalised according to the total useful MS signal (TUS) detected for each sample.

### Chemometric and multivariate statistical modelling

The resulting data set was formatted and exported to MetaboAnalyst version 6.0 software (https://www.metaboanalyst.ca/). A batch effect correction, using the ComBat method, was applied using the ‘other utilities’ feature in MetaboAnalyst. The batch-corrected data set was then normalised to the sample median, log-transformed, and auto-scaled. Principal component analysis (PCA) and partial least squares-discriminant analysis (PLS-DA) were applied to obtain grouping information and significant metabolites, respectively. PLS-DA was used to calculate the VIP values for each variable. These variables were then subjected to univariate analysis using a difference-of-means test (Wilcoxon test). The PLS-DA model was cross-validated to provide estimates of the specificity and sensitivity of the model (Q^2^ and R^2^, respectively). The metabolites with VIP > 1 and *p* < 0.05 were selected as final differential metabolites. Additionally, fold changes (FCs) were calculated to evaluate differences between metabolites in treated compared to untreated cells. The identities of the statistically significant metabolites were confirmed through the Human Metabolome Database (HMDB) (https://hmdb.ca/).

### Pathway mapping

Metabolic pathway analysis was performed using MetaboAnalyst version 6.0 software (https://www.metaboanalyst.ca/). The statistically significant metabolites were used to determine the biological pathways that were altered/disrupted under the given treatment conditions. The built-in eukaryote Homo sapiens (KEGG) pathway library was used as a reference and the hypergeometric test for over-representation analysis and the relative-betweenness centrality for pathway topology analysis were employed as the pathway mapping algorithms.

## Results

### Quality control– batch effect analysis

The severity of any batch effect was assessed and summarised using principal component analyses (PCA). The batch effect assessment was performed on the quality control (QC) samples. No prominent batch effect was visible; however, batch correction was performed but proved insignificant (Supplementary Fig. S1).

### Metabolite profiling of A375 cells after GA113 treatment

**Fig. 2:**
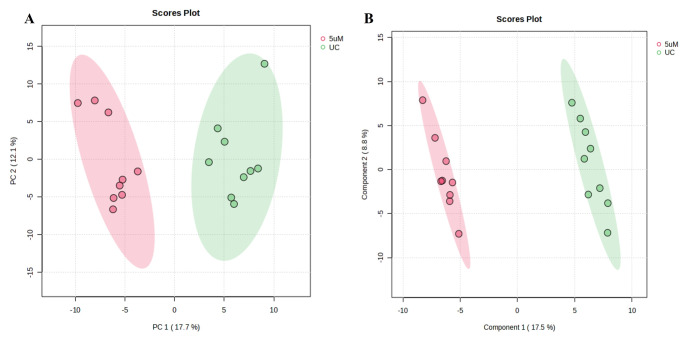
PCA and PLS-DA scores plot of GCXGC-TOF/MS data for A375 cell extracts of untreated control (UC; green) and GA113-treated (5 µM; red) groups. (**A**) The PCA scores plot was computed from PC1 and PC2, explaining 17.7% and 12.1% of the variation in the dataset, respectively. The unsupervised PCA model depicts sample grouping that discriminates between the respective groups. PC1 represents the between-group variation and PC2 represents the within-group variation. (**B**) PLS-DA scores plot (R^2^X = 0.99 and Q^2^ = 0.90) depict the supervised separation between the untreated and treated groups

PCA was done to analyse the intrinsic variation in the GCxGC-TOF/MS dataset (Fig. 2A). The PCA model was derived from principal component 1 (PC1), which accounted for 17.7% of the total variance, and PC2, which accounted for 12.1% of the total variance.

To optimise the observed differences and extract relevant biological information, a supervised partial least squares-discriminant analysis (PLS-DA) model was generated for the treated 5 µM concentration compared to the untreated control (Fig. 2B). The PLS-DA score plots were further validated through cross-validation, yielding robust predictive parameters. The PLS-DA model showed a prediction accuracy of 90% (Q^2^) and a spectral variation (R^2^X) of 99% (Supplementary Fig. S2).

### Variable selection– significantly altered metabolites

A total of 255 metabolites were detected, but only 130 were successfully annotated by spectral comparison with a commercial NIST library. Although the remaining 125 could not be annotated, they were still included in all statistical analyses and classified as ‘unknown’. Because of the classification procedure, some compounds with similar molecular structures, and consequently similar mass spectra, were assigned the same annotation based on the best library match. Retention times and unique mass fragments were examined to distinguish whether these were separate compounds. If confirmed as distinct, the duplicate annotation was retained with an added code and the concentrations were analysed as independent compounds.

**Fig. 3:**
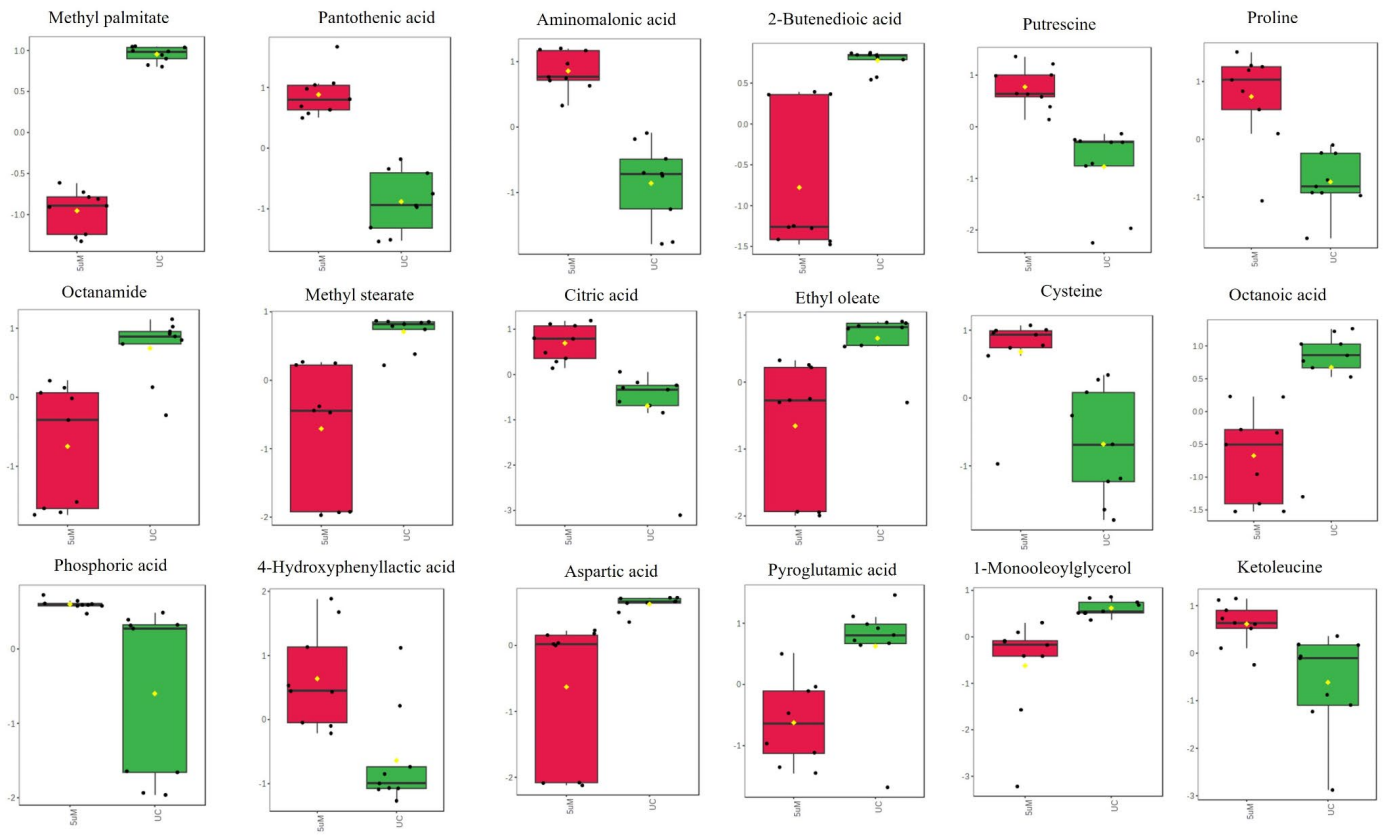
Box plots showing the relative concentrations of significant intracellular metabolites

Treatment with GA113 at 5 µM induced significant changes in 33 metabolites, 19 of which were successfully annotated (Table 2). Notably, several fatty acids, including hexadecanoic acid, octanoic acid, and ethyl oleate, exhibited significant reduction in the treated cells. Conversely, citric acid, proline, cysteine, and phosphoric acid levels were significantly elevated in the A375 cells after GA113 treatment. A quantitative comparison, in the form of box plots of the significant metabolites, is shown in Fig. 3.

### Altered metabolic pathways

**Fig. 4:**
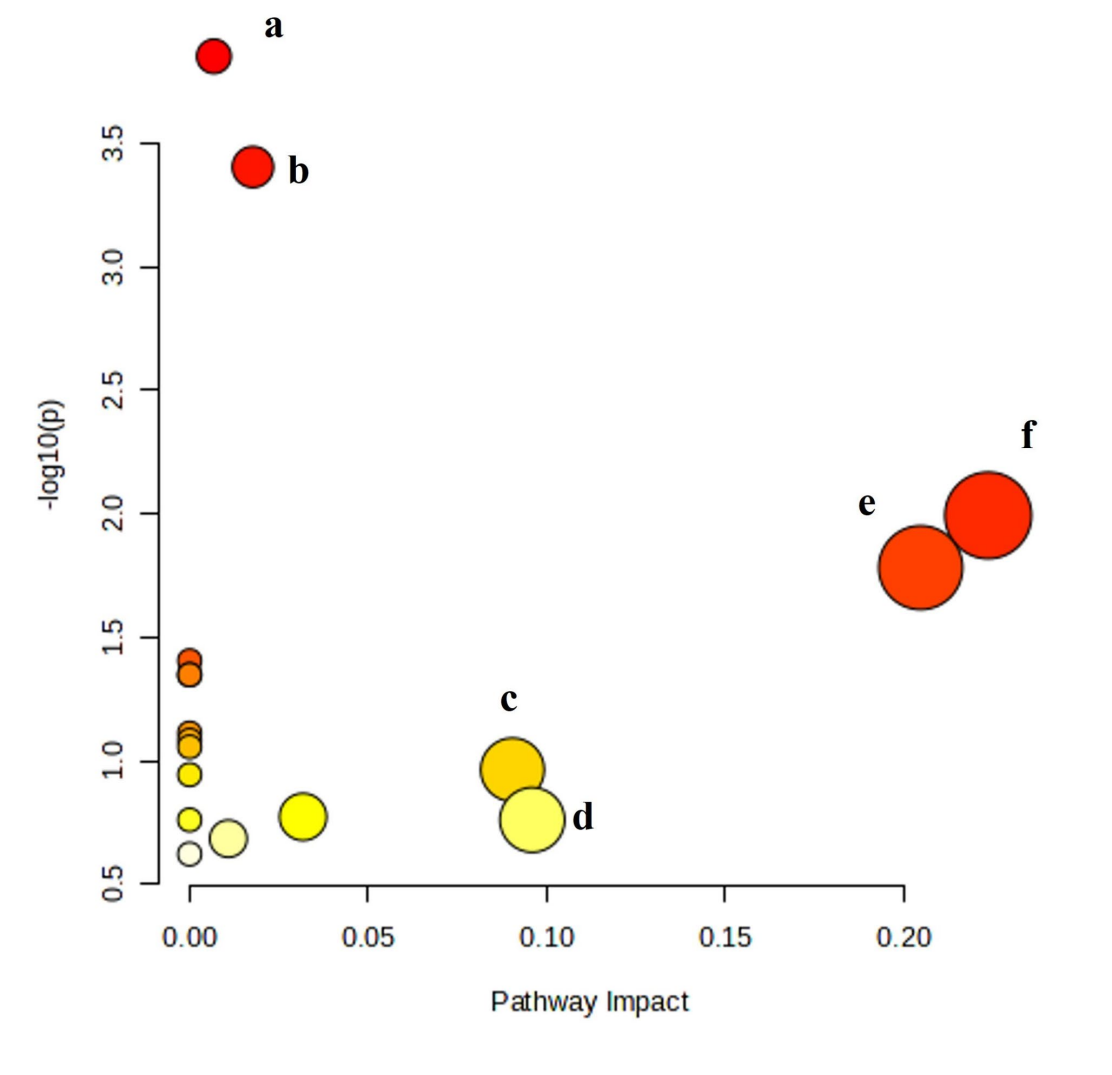
Summary plot for metabolic pathway analysis of 5 µM GA113 treatment in A375 cells. (**a**) Pantothenate and CoA biosynthesis; (**b**) glutathione metabolism; (**c**) citrate cycle; (**d**) cysteine and methionine metabolism; (**e**) arginine and proline metabolism; (**f**) alanine, aspartate, glutamate metabolism

Based on the 19 significantly altered annotated metabolites, metabolic pathway analysis was performed using MetaboAnalyst version 6.0 software (https://www.metaboanalyst.ca/). Metabolic pathways with influence values greater than 0.1 and/or p-value < 0.05 were used as the main metabolic pathways to analyse the potential mechanism of action. Based on the above criteria, the following pathways were considered relevant: pantothenate and coenzyme A (CoA) biosynthesis, glutathione metabolism, citrate cycle, cysteine and methionine metabolism, arginine and proline metabolism, and alanine, aspartate, and glutamate metabolism (Fig. 4).

**Table 2:** Quantitative statistical measures of signatory metabolites discriminating between the GA113 treated (at 5 µM) and untreated A375 cells using GCxGC–TOF/MS

ID	Metabolite	HMDB	Trend	Log2(FC)^a^	*p*-value^b^	VIP value^c^
Var127	Hexadecanoic acid, methyl ester	Methyl palmitate	↓	-7.33	< 0.001	2.36
Var36	Pantothenic acid tritms	Pantothenic acid	↑	1.32	< 0.001	2.18
Var2	2-Aminomalonic acid, N,O, O,-TMS	Aminomalonic acid	↑	2.58	< 0.001	2.12
Var64	Unknown	Unknown	↑	2.29	< 0.001	2.07
Var83	Unknown	Unknown	↓	-2.38	0.001	2.01
Var113	2-Butenedioic acid, (E)-, 2TMS derivative	2-Butenedioic acid	↓	-6.69	0.003	1.92
Var130	Putrescine, 4TMS derivative	Putrescine	↑	2.03	0.003	1.91
Var128	L-Proline, 2TMS derivative	Proline	↑	1.82	0.008	1.82
Var93	Unknown	Unknown	↓	-1.14	0.014	1.76
Var84	Octanamide, N-(2-hydroxyethyl)	Octanamide	↓	-2.33	0.014	1.76
Var144	Methyl stearate	Methyl stearate	↓	-5.46	0.014	1.75
Var161	Unknown	Unknown	↓	-3.34	0.018	1.73
Var7	Citric acid, 4TMS derivative	Citric acid	↑	1.42	0.018	1.72
Var96	Unknown	Unknown	↓	-2.26	0.019	1.70
Var1	(E)-9-Octadecenoic acid ethyl ester	Ethyl oleate	↑	1.85	0.019	1.70
Var16	L-Cysteine, 3TMS derivative	Cysteine	↑	2.12	0.019	1.68
Var50	Unknown	Unknown	↑	1.91	0.020	1.68
Var106	Octanoic acid, 2-dimethylaminoethyl ester	Octanoic acid	↓	-1.63	0.020	1.67
Var65	Unknown	Unknown	↑	1.73	0.021	1.67
Var156	Unknown	Unknown	↓	-3.33	0.022	1.65
Var227	Unknown	Unknown	↑	3.92	0.024	1.63
Var147	9-Octadecenoic acid (Z)-, methyl ester	Ethyl oleate	↓	-4.63	0.025	1.62
Var90	4-Hydroxyphenyllactic acid, 3TMS derivative	4-Hydroxyphenyllactic acid	↑	0.96	0.034	1.58
Var126	L-Aspartic acid, 2TMS derivative	Aspartic acid	↓	-5.43	0.039	1.55
Var86	Unknown	Unknown	↑	1.57	0.039	1.56
Var41	Pyroglutamic acid, TMS derivative	Pyroglutamic acid	↓	-1.33	0.040	1.55
Var77	1-Monooleoylglycerol, 2TMS derivative	1-Monooleoylglycerol	↓	-1.24	0.042	1.54
Var145	Unknown	Unknown	↓	-4.69	0.042	1.54
Var143	Unknown	Unknown	↓	-4.78	0.042	1.52
Var149	2-Ketoisocaproic acid mo-tms	Ketoleucine	↑	1.29	0.042	1.52
Var125	Unknown	Unknown	↓	-5.40	0.043	1.52
Var66	Unknown	Unknown	↑	1.50	0.047	1.50
Var219	Phosphoric acid, 2-(methoxyimino)-3-[(trimethylsilyl)oxy]propyl bis(trimethylsilyl) ester	Phosphoric acid	↑	3.57	0.049	1.48

a) Fold change given as log2 value; (b) p-value corrected for false discovery rate; (c) variables of importance in the projection generated using MetaboAnalyst

## Discussion

We have previously shown that our synthesised bimetallic ruthenium complex (referred to as GA113) exerts an evident cytotoxic effect on the malignant A375 cell line (Engelbrecht et al., 2020; Hussan et al., 2023). In this study, we extended the analysis using a GCxGC-TOF/MS-based metabolomics approach to better understand the cellular response of the A375 cells to GA113. The findings of this study provide evidence of the metabolic reprogramming induced by GA113 treatment in A375 melanoma cells. The identification of 33 signatory metabolites (associated with GA113 treatment) highlights the potential of this bimetallic complex as a therapeutic agent. The pathway analysis revealed significant alterations in critical metabolic pathways, particularly those related to glutathione metabolism, pantothenate and CoA biosynthesis, citrate cycle, cysteine and methionine metabolism, arginine and proline metabolism, and alanine, aspartate, and glutamate metabolism.

### GA113 disrupts oxidative metabolism and energy production

Significant changes in glutathione metabolism and cysteine and methionine metabolism were observed, with notable upregulation of cysteine and phosphate and downregulation of pyroglutamic acid. Glutathione metabolism is closely interconnected with cysteine and methionine metabolism, as these pathways share intermediates and enzymes, and influence cellular redox homeostasis, detoxification, and sulphur metabolism. In this regard, cysteine and pyroglutamic acid play a critical role in cancer metabolism by fuelling cancer cell proliferation, survival, and adaptation to metabolic stress (Lieu et al., 2020; Min et al., 2023; Wei et al., 2021). Both pyroglutamic acid and cysteine are critical components of the γ-glutamyl cycle. Pyroglutamic acid reflects the activity of the cycle, while cysteine availability limits glutathione synthesis (Bachhawat & Yadav, 2018; Stipanuk et al., 2006; Wu et al., 2004). Cancer cells with high oxidative stress can channel pyroglutamic acid directly into glutathione synthesis to maintain redox homeostasis (Gamarra et al., 2019; Tretter et al., 2022). Thus, decreased concentrations of pyroglutamic acid could suggest an imbalance in the cellular redox state, inferring the presence of oxidative damage. Furthermore, increased cysteine concentrations can bolster the cell’s capacity to combat oxidative stress, by increasing the pool of available precursors for glutathione (Wu et al., 2004). Therefore, GA113 could induce apoptosis in the A375 cells through oxidative stress. These observed changes in glutathione metabolism are consistent with our previous findings (Hussan et al., 2023). Unfortunately, we did not detect any changes in glutathione, which would further support the observed changes in glutathione metabolism.

Compared to the untreated cells, the concentrations of pantothenic acid and citric acid (citrate) increased, while 2-butenedioic acid (also known as fumarate) decreased in the GA113-treated cells. Citrate is a precursor to acetyl-CoA, which is involved in energy production and lipid biosynthesis (Icard et al., 2012; Zhao et al., 2022). Increased pantothenic acid can lead to increased levels of coenzyme A (CoA), which supports the conversion of citrate to acetyl-CoA for fatty acid synthesis (Czumaj et al., 2020; Leonardi & Jackowski, 2016). Pantothenic acid is also a precursor of CoA, which is involved in several metabolic pathways, including the citrate cycle, fatty acid synthesis, and acetylation reactions (Czumaj et al., 2020; Leonardi & Jackowski, 2016). Cancer cells often exhibit altered metabolism, including increased lipid biosynthesis for membrane production and energy storage. Pantothenic acid, through its role in CoA synthesis, supports fatty acid synthesis by providing acetyl-CoA. Thus, increased pantothenic acid could be a response to the increased energy and biosynthetic demands placed on the treated A375 cells. Thus, the upregulation of pantothenic acid can suggest that the cells are trying to cope with oxidative stress, DNA damage, and disruption of the cell cycle induced by the treatment. In addition, the observed increase in citrate levels could be a sign of enhanced mitochondrial activity to support energy production (Martínez-Reyes & Chandel, 2020; Zara et al., 2022). Taken together, the combination of decreased 2-butenedioic acid (fumarate) and increased citrate suggests a disrupted citrate cycle, potentially caused by impaired downstream flux, while increased levels of pantothenic acid suggest a shift toward oxidative metabolism and energy production in response to GA113 treatment.

### GA113 disrupts amino acid biosynthesis

Metabolic pathway analysis showed significant changes in arginine and proline metabolism, as well as alanine, aspartate, and glutamate metabolism. These pathways are crucial in cellular metabolism and are tightly linked in the context of amino acid biosynthesis and catabolism (Chandel, 2021; Reitzer, 2004). Previous studies have highlighted the role of amino acid biosynthesis in cancer, particularly in supporting nucleotide biosynthesis and maintaining redox homeostasis, which is vital for sustaining cell proliferation and combating oxidative stress (Wei et al., 2021). The observed shifts in amino acid levels (proline, aspartic acid, aminomalonic acid, and ketoleucine) further reinforce the hypothesis that GA113 exerts its anticancer effects by targeting metabolic networks fundamental for melanoma cell survival.

### GA113 disrupts lipid metabolism and fatty acid synthesis

To meet energy demands, cancer cells can redirect metabolic dependencies through increased lipid and fatty acid synthesis, which supports rapid proliferation (Butler et al., 2020). In addition, when cancer cells are treated with an apoptotic inducer, fatty acid levels can decrease due to several factors related to the ability of cancer cells to synthesise or acquire fatty acids (Gnocchi et al., 2021). In this study, the concentrations of several fatty acids, including methyl palmitate (a methyl ester of palmitic acid), methyl stearate (a methyl ester of stearic acid), ethyl oleate, octanoic acid, 1-monooleoylglycerol, and octanamide (a derivative of octanoic acid), were significantly decreased in the treated cells compared to the untreated cells (Table 2). The decrease in the abovementioned metabolites suggests a substantial disruption in lipid metabolism and fatty acid biosynthesis, both of which are crucial for maintaining the structural integrity of cell membranes and supporting bioenergetic needs (Duarte et al., 2013; Lumaquin-Yin et al., 2023; Pellerin et al., 2020).

Methyl esters can act as markers of lipid metabolism and oxidative stress. Thus, the observed decrease in methyl palmitate and methyl stearate can reflect altered lipid turnover or oxidative degradation. The decreased levels of octanoic acid and octanamide can suggest disrupted fatty acid β-oxidation, as octanoic acid is a key intermediate in mitochondrial energy metabolism (Altinoz et al., 2020; Rial et al., 2018). Ethyl oleate and 1-monooleoylglycerol are derivatives of oleic acid, which is often involved in lipid signalling and energy storage (Koundouros & Poulogiannis, 2020). Thus, the observed changes in ethyl oleate and 1-monooleoylglycerol suggest that the GA113 complex can induce apoptosis by influencing the biological processes related to membrane synthesis and structural integrity.

Although our study successfully highlighted potential drug targets, some key limitations should be noted. For instance, we only analysed the treated A375 cells at a single time point (24 h after treatment). Metabolism is inherently time-dependent, with metabolites fluctuating in response to cellular processes, environmental changes, or treatment effects. By focussing on a single time point, the study could have missed critical phases of metabolic activity. This limitation restricts the ability to distinguish transient changes from sustained metabolic shifts, making it challenging to fully understand the mechanisms underlying observed effects or to identify causal relationships between treatment and metabolic outcomes. Nonetheless, the findings of this study highlight several crucial areas for future research. For example, to further validate our findings, studies on the metabolic effect of GA113 in non-cancerous cells (e.g., HEK293) are required to determine and compare the mechanism of action in both malignant and non-malignant cell lines. Such studies will contribute to the identification of specific targets of GA113 in both cell lines. To further establish the therapeutic potential of GA113, in vivo studies are essential to validate the metabolic effects observed in A375 melanoma cells and to assess the pharmacokinetics, pharmacodynamics, and toxicity profile in an animal model.

## Conclusion

In this study, we used GCxGC-TOF/MS to analyse metabolite alterations in A375 cells treated with GA113 using univariate and multivariate statistical methods. Our findings suggest that exposure to GA113 can potentially lead to oxidative stress at the cellular level and disrupt amino acid, lipid and energy metabolism. These findings confirm the value of metabolomics in elucidating the potential mode of action of anticancer agents. Given the dynamic nature of cancer metabolism, leveraging metabolomics approaches can facilitate the identification of biomarkers that could guide therapeutic decision making and patient stratification in clinical settings.

## Electronic supplementary material

Below is the link to the electronic supplementary material.

Supplementary Material 1

## Data Availability

Data is provided within the manuscript or supplementary information files. Any other data will be made available upon request.
