# Supplementary material for: Metabolic reprogramming in malignant A375 cells treated with a ruthenium (II) complex: insights from GCxGC-TOF/MS metabolomics
Source: Metabolomics. 2025 Jan 20;21(1):18. doi: 10.1007/s11306-025-02221-7 (PMC11825624; doi:10.1007/s11306-025-02221-7)
Supplement: Supplementary file 1 — Supplementary Material 1 [file 11306_2025_2221_MOESM1_ESM.docx]

**Metabolic reprogramming in malignant A375 cells treated with a ruthenium (II) complex: Insights from GCxGC-TOF/MS metabolomics**

Francis Adu-Amankwaah^2^, Ayesha Hussan^1^, Gershon Amenuvor^3^, Vuyo Mavumengwana^2^ and Lungile Sitole*^1^

^1^Department of Biochemistry, Faculty of Science, University of Johannesburg, Johannesburg, 2006, South Africa.

^2^South African Medical Research Council Centre for Tuberculosis Research, Division of Molecular Biology and Human Genetics, Faculty of Medicine and Health Sciences, Stellenbosch University, Stellenbosch, 7505, South Africa.

^3^Department of Chemistry, Faculty of Science and Computational Sciences, Kwame Nkrumah University of Science and Technology, Kumasi, Ghana.

**Address correspondence to this author at the Biochemistry Department, University of Johannesburg, Johannesburg 2006, South Africa. Tel: +27115592984; E-mail:* [*lsitole@uj.ac.za*](mailto:lsitole@uj.ac.za)

**Supplementary Material**


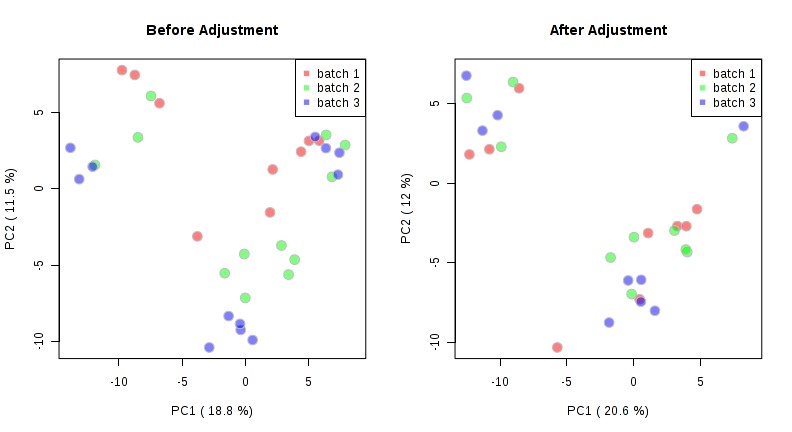


**Supplementary Fig. S1** Principal component analysis (PCA) before and after batch correction indicating no severe batch effect. The cumulative variation explained by the first two principal components (PCs) was 30.3 % before batch effect correction. The cumulative variation explained by the first two principal components (PCs) was 32.6 % after batch effect correction.


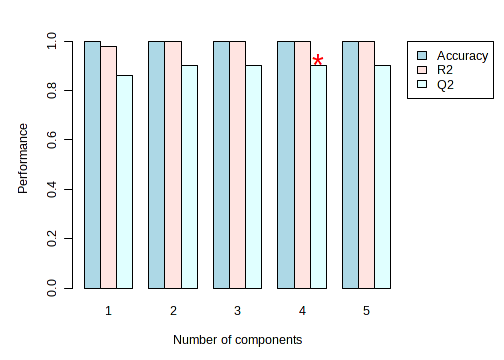


**Supplementary Fig. S2** Validation of the PLS-DA model. Performance measures of the PLS-DA model were assessed by prediction accuracy, R^2^ and Q^2^ values. The fourth component, indicated by the red *, best classifies the model.
